# Ginsenoside Rd promotes the expression of acetylated α-tubulin and FOXJ1 in human bronchial epithelial cells

**DOI:** 10.1007/s11418-026-02084-9

**Published:** 2026-09-23

**Authors:** Kouta Noriyama, Seikou Nakamura, Nobuyo Tamiya, Yuki Toda, Shigekuni Hosogi, Eishi Ashihara

**Affiliations:** 1 Laboratory of Clinical and Translational Physiology, Kyoto Pharmaceutical University, 5 Nakauchi-cho, Misasagi, Yamashina-ku, Kyoto, 607-8414 Japan; 2 Laboratory of Pharmacognosy, Kyoto Pharmaceutical University, 1 shichono-cho, Misasagi, Yamashina-ku, Kyoto, 607-8412 Japan; 3 Pulmonary Medicine, Rakuwakai Otowa Hospital, 2 chinji-cho, Otowa, Yamashina- ku, Kyoto, 607-8062 Japan; 4 Department of Pulmonary Medicine, Graduate School of Medical Science, Kyoto Prefectural University of Medicine, 465 Kajii-cho, Kawaramachi-Hirokoji, Kamigyo- ku, Kyoto, 602-8566 Japan; 5 Present Address: Kyoto Red Cross Blood Center, Nakajimakitanokuchi-cho, Fushimi-ku, Kyoto, 612-8451 Japan

**Keywords:** Ciliated cell, Ginsenoside Rd, Acetylated α-tubulin, Ciliary beat frequency

## Abstract

Mucociliary clearance is an airway defense mechanism driven by the coordinated function of mucus and motile cilia. Although ciliary beat frequency (CBF) reflects the motile function of ciliated cells, the generation and maintenance of ciliated cells are also essential for sustained airway epithelial defense. Although short-term treatment with ginsenoside Rd (Rd) increases CBF, its long-term effects on the differentiation of human airway epithelial cells remain unclear. We investigated the effects of Rd on primary normal human bronchial epithelial cells cultured under air–liquid interface conditions. On day 10, Rd increased the percentage of acetylated α-tubulin (Ac-α-tubulin)-positive cells, whereas the percentage of MUC5AC-positive cells remained unchanged. Rd promoted the time-dependent increase in Ac-α-tubulin-positive cells, while LDH release or total cell number were not significantly altered. Long-term Rd treatment did not affect baseline CBF. Furthermore, perfusion with Rd increased CBF independent of differentiation conditions. These observations suggest that the increase in Ac-α-tubulin-positive cells by long-term Rd treatment and the increase in CBF by short-term Rd treatment may be functionally separable. The increase in the percentage of Ac-α-tubulin-positive cells was suppressed by ERβ inhibitors and CREB-dependent transcription inhibitors. Rd also increased the expression of *FOXJ1* mRNA transcripts. These findings suggest that Rd may modulate the expression of ciliated cell markers, such as acetylated α-tubulin and FOXJ1, and ciliary motility in human airway epithelial cells.

**Graphical abstract:**

**Figure:**
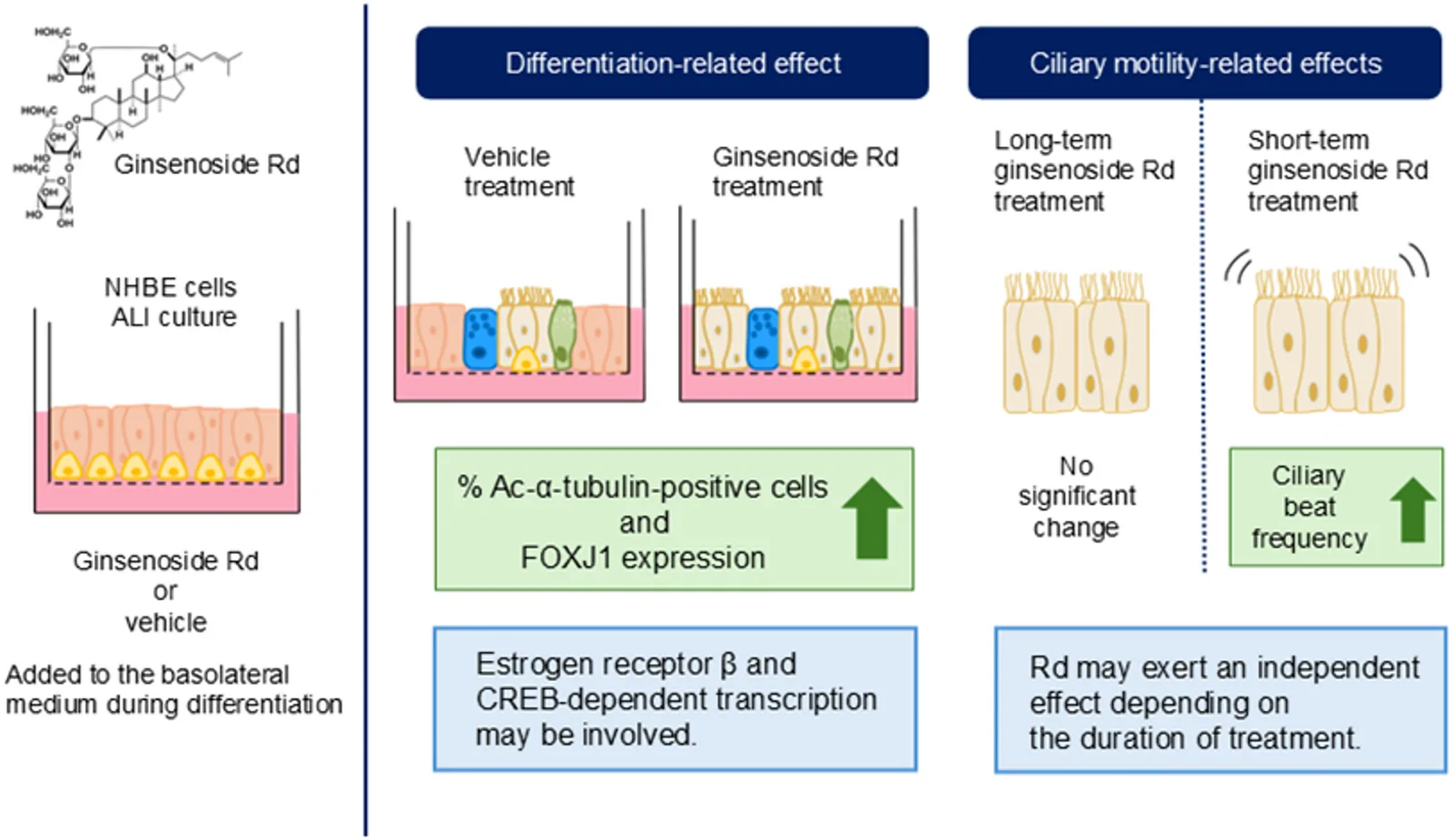

**Supplementary Information:**

The online version contains supplementary material available at https://doi.org/10.1007/s11418-026-02084-9.

## Introduction

Mucociliary clearance (MCC) in the airways is an important defense mechanism against inhaled pathogens and environmental particles [1–3]. MCC depends on the coordinated interaction between mucus produced by secretory cells and the motile cilia of ciliated epithelial cells [4]. Ciliary beat frequency (CBF) is widely used as an indicator of ciliary function and MCC [5–8]. Both increased CBF and differentiation into ciliated cells are essential for the long-term maintenance and activation of MCC.

A multistep transcriptional program tightly regulates ciliated cell differentiation in the airway epithelium. Notch signaling contributes to the balance between secretory and ciliated epithelial cell fates [9–11]. Differentiation into ciliated cells is driven by the induction of geminin coiled-coil domain-containing protein 1 and multiciliate differentiation and DNA synthesis-associated cell cycle (MCIDAS), followed by the upregulation of cyclin O (CCNO) and forkhead box J1 (FOXJ1) expression [11]. Among these factors, FOXJ1 is regarded as a master transcription factor for ciliated cell differentiation and regulates the expression of genes involved in ciliary structural components, basal body formation, and motile cilia function.

In addition to these lineage-regulatory mechanisms, cAMP-responsive transcriptional pathways may also influence differentiation into ciliated cells. cAMP response element binding protein 1 (CREB1) is a transcription factor activated downstream of cAMP signaling, and Creb1 deficiency has been reported to delay respiratory epithelial differentiation during murine lung development, including the appearance of Foxj1-positive ciliated epithelial cells [12]. However, whether CREB-dependent transcription contributes to the pharmacological regulation of differentiation into ciliated cells in human airway epithelial cells remains unclear.

Ginsenoside Rd (Rd) is a bioactive ginsenoside derived from *Panax ginseng*. Some ginsenosides have been reported to exert various pharmacological effects, including estrogen-like activity [13, 14]. We recently reported that Rd and 17β-estradiol increase CBF in murine airway ciliated cells through estrogen receptor (ER) β [6, 15]. However, the long-term effects of Rd on airway epithelial differentiation remain unclear. ERβ signaling has been implicated in differentiation into ciliated cells in porcine fallopian tube and human endometrial organoids [16–18], but its role in the airway has not been fully elucidated. ERβ is also broadly expressed in the human respiratory tract, with limited sex-related differences [19, 20]. Based on these findings, we hypothesized that ERβ-associated signaling may promote differentiation into ciliated cells in the human airway epithelium. Therefore, the present study aimed to investigate whether Rd affects the expression of acetylated α-tubulin and FOXJ1, which are ciliated cell markers, and CBF using primary normal human bronchial epithelial (NHBE) cells cultured under air–liquid interface (ALI) conditions.

## Materials and methods

### Expansion of NHBE cells

NHBE cells were obtained from a single donor (male, 18 years old, Caucasian, no history of smoking, Mycoplasma-negative, and negative for hepatitis B virus, hepatitis C virus, and human immunodeficiency virus-1 and − 2) from the American Type Culture Collection (ATCC, PCS-300-010, Manassas, VA, USA). The culture medium was Airway Epithelial Cell Basal Medium (PCS-300-030, ATCC) supplemented with Bronchial Epithelial Cell Growth Kit (PCS-300-040, ATCC), 100 U/mL penicillin, and 100 µg/mL streptomycin (FUJIFILM Wako Pure Chemical Corporation, Osaka, Japan). The medium was pre-equilibrated for 30 min at 37℃ in a 5% CO₂ incubator before use. NHBE cells were seeded at 5.0 × 10³ cells/cm² in a 25-cm² flask (#430639, Corning, NY, USA) and defined as passage 1. Cells were passaged at 70–80% confluence. To detach the cells, cells were treated with 0.025% trypsin and 0.1 mM ethylenediaminetetraacetic acid (FUJIFILM Wako Pure Chemical Corporation) and subsequently neutralized with trypsin inhibitor (Nacalai Tesque, Kyoto, Japan). All solutions were sterilized by filtration through a 0.22 μm filter (Sigma-Aldrich, St. Louis, MO, USA). Cells at passages 2–4 were cryopreserved at 5.0 × 10⁵ cells/vial using STEM-CELLBANKER^®^ GMP grade (ZENOGEN PHARMA, Fukushima, Japan) and used for the experiments.

### ALI culture

NHBE cells were seeded at 1.0 × 10⁴ cells/well on 24-well transwell inserts (#3470, Corning). The cells were cultured in PneumaCult-Ex medium (STEMCELL Technologies, Vancouver, BC, Canada) under submerged conditions until they reached 100% confluence. After confluence was reached, the apical medium was removed, and the basolateral medium was replaced with PneumaCult-ALI medium (STEMCELL Technologies). The medium was changed every 2 days. For reagent treatments, reagents were added to the basolateral medium, and the final dimethyl sulfoxide (DMSO) concentration was maintained at 0.1%.

### Solutions and chemicals

**Fig. 1:**
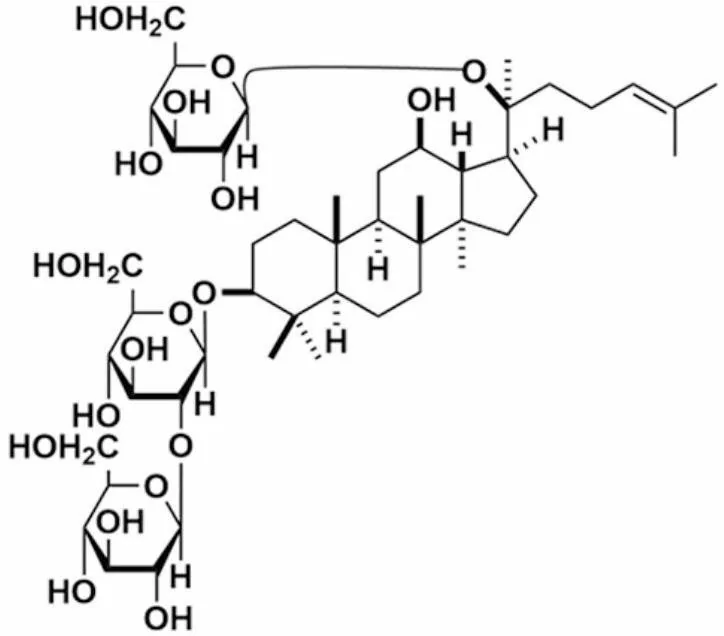
The chemical structure of Rd. Ginsenoside Rd, a bioactive saponin derived from *Panax ginseng*, is a protopanaxadiol-type ginsenoside. It consists of a dammarane-type tetracyclic triterpene aglycone with sugar moieties attached at the C-3 and C-20 positions

The composition of the control solution has been described previously [15, 21]. DMSO, albumin from bovine serum (BSA), and forskolin were purchased from FUJIFILM Wako Pure Chemical Corporation. Hoechst 33342 was purchased from Thermo Fisher Scientific Inc. (Waltham, MA, USA). Rd (Fig. 1) was purchased from Tokyo Chemical Industry Co., Ltd (Tokyo, Japan). PHTPP, G15, and compound 3i were purchased from Selleck Biotech (Kanagawa, Japan).

### Immunofluorescence staining and quantification of marker-positive cells

NHBE cells were fixed with 4% paraformaldehyde for 12 min, and unreacted aldehyde groups were quenched with PBS containing 0.2 M glycine for 20 min. The cells were then blocked with PBS containing 5% BSA for 1 h at room temperature and incubated with primary antibodies overnight at 4℃. Subsequently, secondary antibodies were applied for 1 h at room temperature. Hoechst 33342 was added together with the secondary antibodies to counterstain the nuclei. Immunolabeling signals were captured using an all-in-one BZ-X800 microscope (Keyence Corporation, Osaka, Japan) or a confocal laser scanning microscope (LSM800; Carl Zeiss, Oberkochen, Germany). The antibodies used in this study are listed in Supplementary Information (SI) 1. For each transwell insert, the entire membrane area was imaged and analyzed. To assess differentiation, we examined the expression of acetylated α-tubulin (Ac-α-tubulin), a ciliated cell marker, and mucin 5, subtype AC (MUC5AC), a mucus-secreting cell marker. The percentage of Ac-α-tubulin-positive or MUC5AC-positive cells was calculated as the number of Ac-α-tubulin-positive or MUC5AC-positive cells divided by the number of Hoechst 33342-positive cells across the entire transwell insert, multiplied by 100. These experiments were performed using five independent inserts per condition, and images from all inserts were analyzed.

### Measurement of CBF

The method used for measuring CBF was reported previously [15]. Differentiated airway epithelial ciliated cells were placed on Cell-Tak (Becton Dickinson Labware, Bedford, MA, USA.) coated coverslip, and the coverslip with cells was set in a micro-perfusion chamber (20 µL) maintained at 37℃. The ciliary beating was observed using an inverted light microscope (IX71; OLYMPUS, Tokyo, Japan) connected to a high-speed camera (HAS-U2; DITECT Ltd., Tokyo, Japan). The chamber was perfused with control solution aerated with a gas mixture of 95% O_2_ and 5% CO_2_ at a constant rate of 200 µL/min. To measure CBF, images were captured at 500 frames/s. CBF was measured using image analysis program (DippMotion 2D, DITECT Ltd.). CBF at 5 min before reagent treatment (CBF_− 5_) was used as the baseline value. %CBF change was defined as follows: [(CBF after 20 min - baseline CBF) / baseline CBF] × 100. Each experiment was performed using five coverslips.

### Experimental design for CBF measurements in long-term and additional short-term Rd treatments

NHBE cells were cultured in ALI conditions for 21 days. Every two days, the medium was replaced with DMSO as a vehicle or 10 µM Rd. The inserts were separated from the transwells 24 h after the last medium change, and CBF measurements were performed. In both the vehicle-treated and Rd-treated culture groups, CBF was measured using a 2 × 2 experimental design consisting of the vehicle-perfused group and the Rd-perfused group, respectively. The data were analyzed using a two-way ANOVA with two factors: culture conditions and perfusion conditions. Tukey’s multiple comparison test was used for post hoc analysis.

### Reverse transcription-quantitative PCR (RT-qPCR)

RNA was purified using the NucleoSpin RNA kit (Takara Bio Inc., Kusatsu, Japan) and subjected to reverse transcription using the ReverTra Ace^®^ qPCR RT kit (TOYOBO CO., LTD., Osaka, Japan) to produce cDNA. mRNA expression levels of human *B2M*, *FOXJ1*, *MCIDAS*, *CCNO*, and *HES1* were measured by real-time PCR using Brilliant III Ultra-Fast SYBR Green QPCR Master Mix (Agilent Technologies, Santa Clara, CA, USA) and the Thermal Cycler Dice Real Time System II (Takara Bio Inc.). The gene-specific primers used in this study are listed in SI 2. RT-qPCR experiments were also performed using RNA obtained from five independent inserts.

### Statistical analysis

Statistical analyses were performed using GraphPad Prism 9 (GraphPad Software Inc., San Diego, CA, USA). The data were obtained from five independent cell experiments conducted with a single donor and are presented as the mean ± standard error of the mean (SEM). Comparisons between two groups were performed using unpaired or paired Student’s t-test, as appropriate. For comparisons among multiple groups, one-way analysis of variance (ANOVA) followed by Dunnett’s and Tukey’s multiple comparison test was used, as appropriate. Two-way ANOVA was performed to analyze experiments involving two independent factors, followed by Tukey’s multiple comparison test as necessary. Quadratic regression analysis was used to evaluate nonlinear concentration–response relationships, and linear trend analysis was performed across the indicated concentration range. Simple linear regression was used to analyze time-course data where indicated. Differences were considered statistically significant at *p* < 0.05.

## Results

### Validation of airway epithelial differentiation and ciliary motility in NHBE cells

**Fig. 2:**
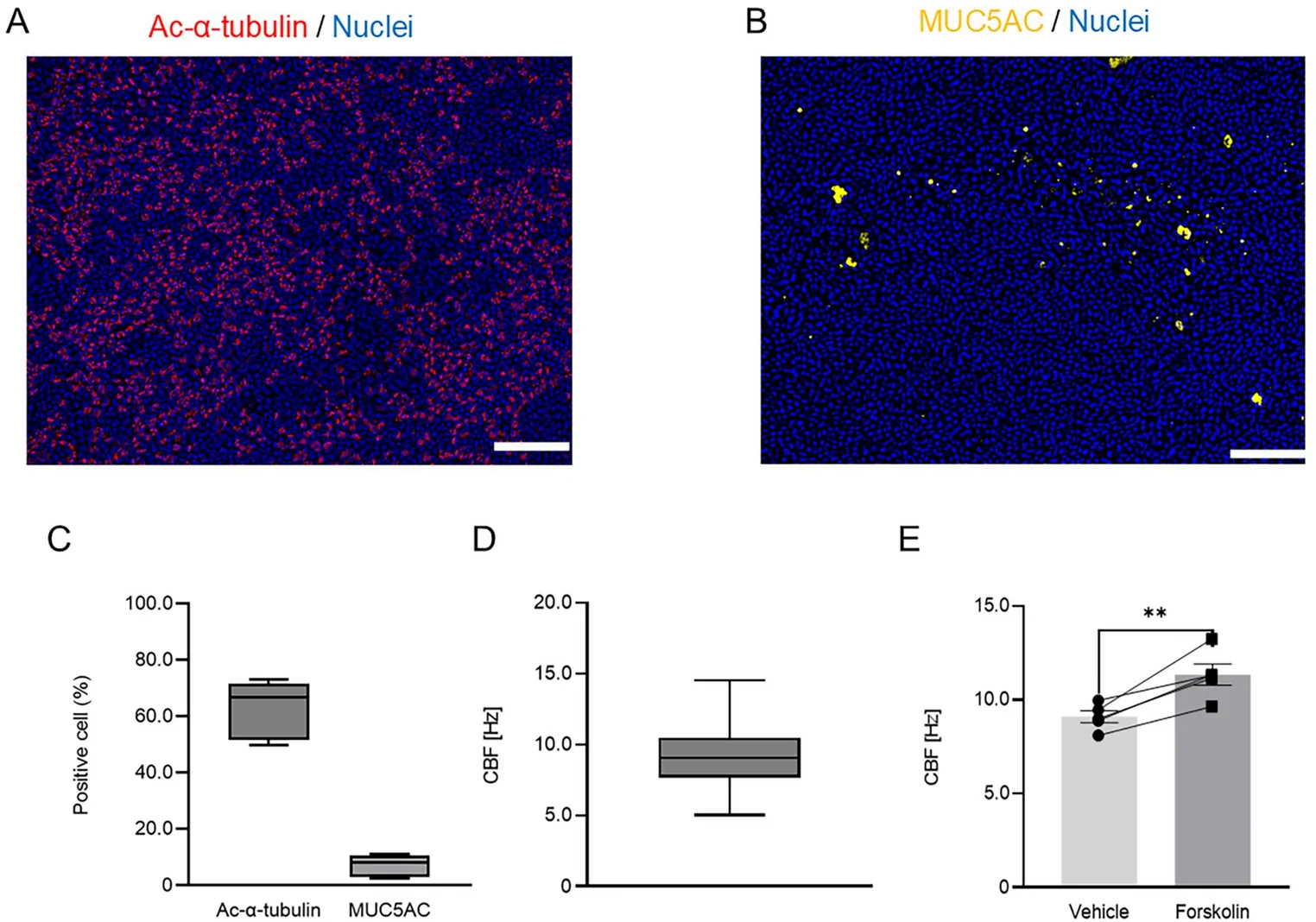
Validation of differentiation and ciliary motility in NHBE cells cultured under ALI conditions. **A**, **B** Representative immunofluorescence images of NHBE cells cultured for 21 days under ALI conditions. These images show nuclei stained with Hoechst 33342 (blue) and staining for (A) Ac-α-tubulin (red) or (B) mucin 5, subtype AC (MUC5AC; yellow). **C** Quantification of the percentages of Ac-α-tubulin-positive and MUC5AC-positive cells. **D** Measurement of baseline ciliary beat frequency (CBF) of ciliated cells differentiated from NHBE cells on day 21 of ALI culture. **E** Changes in CBF after 20 min of forskolin (10 µM) administration. Data are presented as the mean ± SEM of five independent experiments. Data were analyzed using paired Student’s t-test. Statistical significance is indicated as **p < 0.01. All immunofluorescence images were acquired using a 20× objective lens. Scale bars: 100 μm

To validate the suitability of NHBE cells cultured under ALI conditions for the present study, we cultured NHBE cells under ALI conditions for 21 days. Immunofluorescence staining confirmed the expression of Ac-α-tubulin, a ciliated cell marker, and MUC5AC, a marker of mucus-producing secretory cells. The percentages of Ac-α-tubulin-positive and MUC5AC-positive cells were 62.7 ± 4.7% and 7.1 ± 1.7%, respectively (Fig. 2A–C, and SI 3 A, B), which were broadly consistent with previous reports on normal airway epithelium [22, 23]. The CBF of differentiated ciliated cells was 9.1 ± 1.8 Hz (Fig. 2D). Forskolin, an adenylyl cyclase activator that stimulates cAMP signaling, was treated as a positive control for CBF-increasing agents. Forskolin (10 µM) significantly increased CBF when measured 20 min after treatment, with an absolute increase of 2.2 ± 0.1 Hz and a relative increase of 24.4 ± 4.3% from baseline (Fig. 2E). These results indicate that NHBE cells cultured under ALI conditions developed airway epithelial features and retained functional pharmacological responsiveness, supporting the suitability of this model for the present study.

### Rd modulates the percentage of Ac-α-tubulin-positive cells

**Fig. 3:**
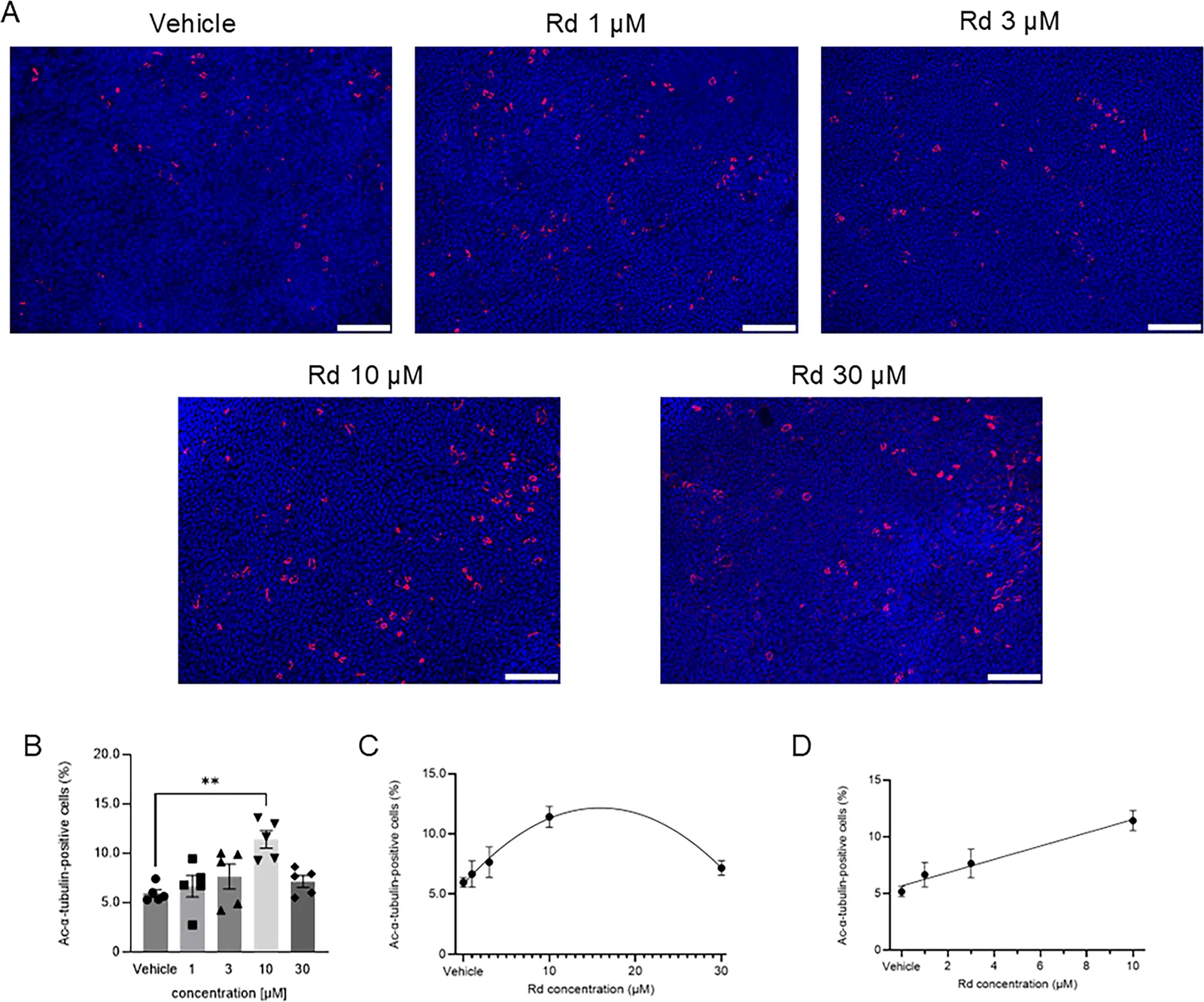

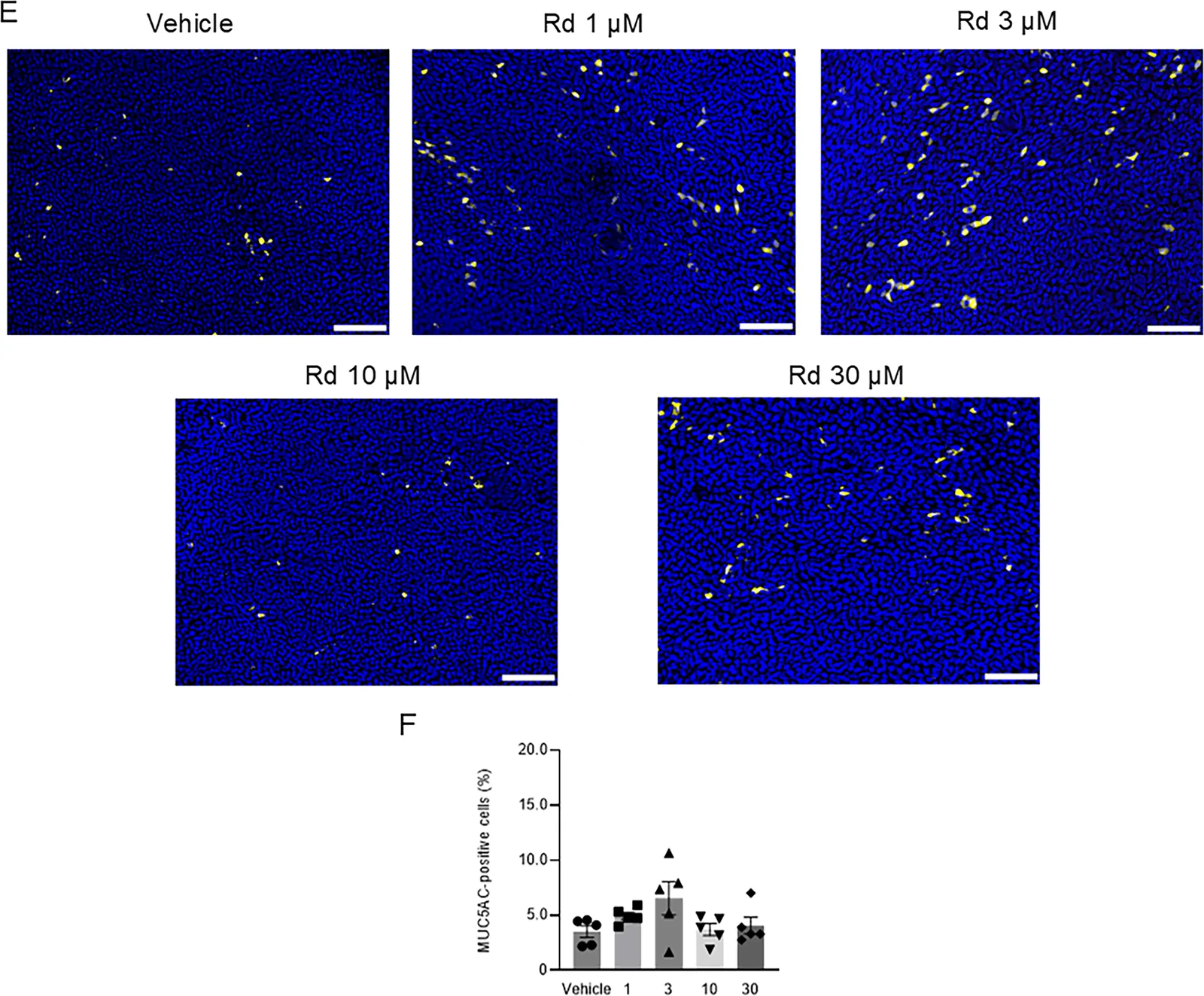
Concentration-dependent effects of Rd on Ac-α-tubulin-positive and MUC5AC-positive cells. **A**, **E** Representative immunofluorescence images of NHBE cells treated with vehicle or 1, 3, 10, or 30 µM Rd from the initiation of ALI culture for 10 days. These images show nuclei stained with Hoechst 33342 (blue) and staining for (A) Ac-α-tubulin (red) or (E) MUC5AC (yellow). **B**,** F** Quantification of the percentage of (B) Ac-α-tubulin-positive or (F) MUC5AC-positive cells. **C** The effects of vehicle and 1, 3, 10, and 30 µM Rd on the percentage of Ac-α-tubulin-positive cells were plotted using a quadratic regression curve. **D** Linear trend analysis across the vehicle, 1, 3, and 10 µM Rd. The slope was significantly positive (*p* = 0.001). Data are presented as the mean ± SEM of five independent experiments. Data were analyzed using one-way ANOVA followed by Dunnett’s multiple comparison test. Statistical significance is indicated as **p < 0.01. All immunofluorescence images were acquired using a 20× objective lens. Scale bars: 100 μm

To investigate whether Rd affects NHBE cell differentiation, cells were treated with Rd at 1, 3, 10, or 30 µM for 10 days beginning at the initiation of ALI culture. Rd significantly increased the percentage of Ac-α-tubulin-positive cells at 10 µM, whereas no significant differences were observed at 1, 3, or 30 µM compared with vehicle-treated cells (Fig. 3A, B, and SI 4A). The percentage of Ac-α-tubulin-positive cells was lower at 30 µM than at 10 µM (Fig. 3C). In addition, linear trend analysis across the vehicle, 1, 3, and 10 µM range showed a significant positive trend (*p* = 0.001), suggesting that Rd increased the percentage of Ac-α-tubulin-positive cells within this lower concentration range (Fig. 3D). In contrast, Rd did not significantly affect the percentage of MUC5AC-positive cells (Fig. 3E, F, and SI 4B). Taken together, these results suggest that Rd may modulate the percentage of Ac-α-tubulin-positive cells, with the maximal effect observed at 10 µM.

### Rd promotes the time-dependent increase in the percentage of Ac-α-tubulin-positive cells

**Fig. 4:**
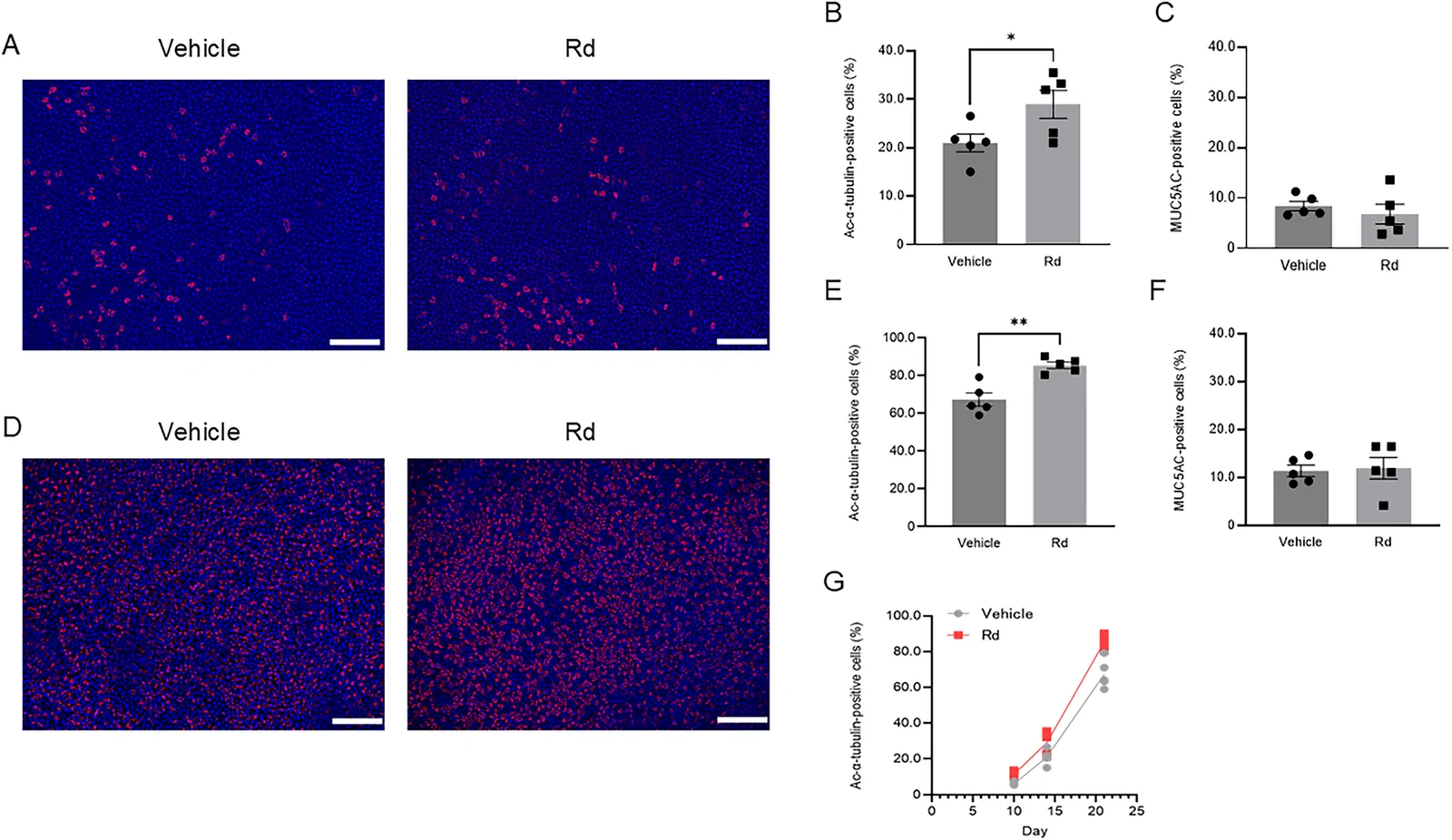
Rd promotes the time-dependent increase in Ac-α-tubulin-positive cells. **A**, **D** Representative immunofluorescence images of NHBE cells treated with vehicle or 10 µM Rd from the initiation of ALI culture for (A) 14 days or (D) 21 days. These images show nuclei stained with Hoechst 33342 (blue) and staining for Ac-α-tubulin (red). **B**, **E** Quantification of the percentage of Ac-α-tubulin-positive cells on (B) day 14 and (E) day 21. **C**, **F** Quantification of the percentage of MUC5AC-positive cells on (C) day 14 and (F) day 21. **G** Time-course analysis of the percentage of Ac-α-tubulin-positive cells on days 10, 14, and 21 of ALI culture. Data are presented as the mean ± SEM of five independent experiments. Data were analyzed using an unpaired Student’s t-test or two-way ANOVA with culture duration and Rd treatment as factors. Statistical significance is indicated as *p < 0.05 and **p < 0.01. All immunofluorescence images were acquired using a 20× objective lens. Scale bars: 100 μm

To investigate the effects of long-term Rd treatment on airway epithelial differentiation, we analyzed NHBE cells cultured under ALI conditions on days 14 and 21. On day 14, Rd significantly increased the percentage of Ac-α-tubulin-positive cells (Fig. 4A, B, and SI 5A), but did not affect the percentage of MUC5AC-positive cells (Fig. 4C and SI 5B). Similarly, on day 21, Rd significantly increased the percentage of Ac-α-tubulin-positive cells (Fig. 4D, E, and SI 5C) without altering the percentage of MUC5AC-positive cells (Fig. 4F and SI 5D). These results suggest that Rd may promote the time-dependent increase in the percentage of Ac-α-tubulin-positive cells. Therefore, we analyzed the time-dependent changes in the percentage of Ac-α-tubulin-positive cells on days 10, 14, and 21. This analysis showed that both culture duration and Rd treatment significantly affected the percentage of Ac-α-tubulin-positive cells, with a significant interaction between these factors (Fig. 4G). These findings suggest that Rd influenced the time-dependent increase in Ac-α-tubulin-positive cells. Consistent with this interaction, slope analysis showed a significantly greater rate of increase in the percentage of Ac-α-tubulin-positive cells in the Rd-treated group than in the vehicle group (Vehicle: 5.7%/day; Rd: 6.9%/day; SI 6A). To further evaluate whether long-term Rd treatment caused cytotoxicity, we assessed lactate dehydrogenase (LDH) release and the number of Hoechst 33342-positive cells on day 21. Rd did not significantly alter LDH release or the number of Hoechst 33342-positive cells, indicating that Rd did not cause detectable cytotoxicity or alter the total cell number under these conditions (SI 6B, C). These results also suggest that the increase in Ac-α-tubulin-positive cells following Rd treatment was not attributable to changes in total cell number, but rather reflected an increased proportion of Ac-α-tubulin-positive cells among the cultured cells.

### Effects on CBF according to the duration of Rd treatment

**Fig. 5:**
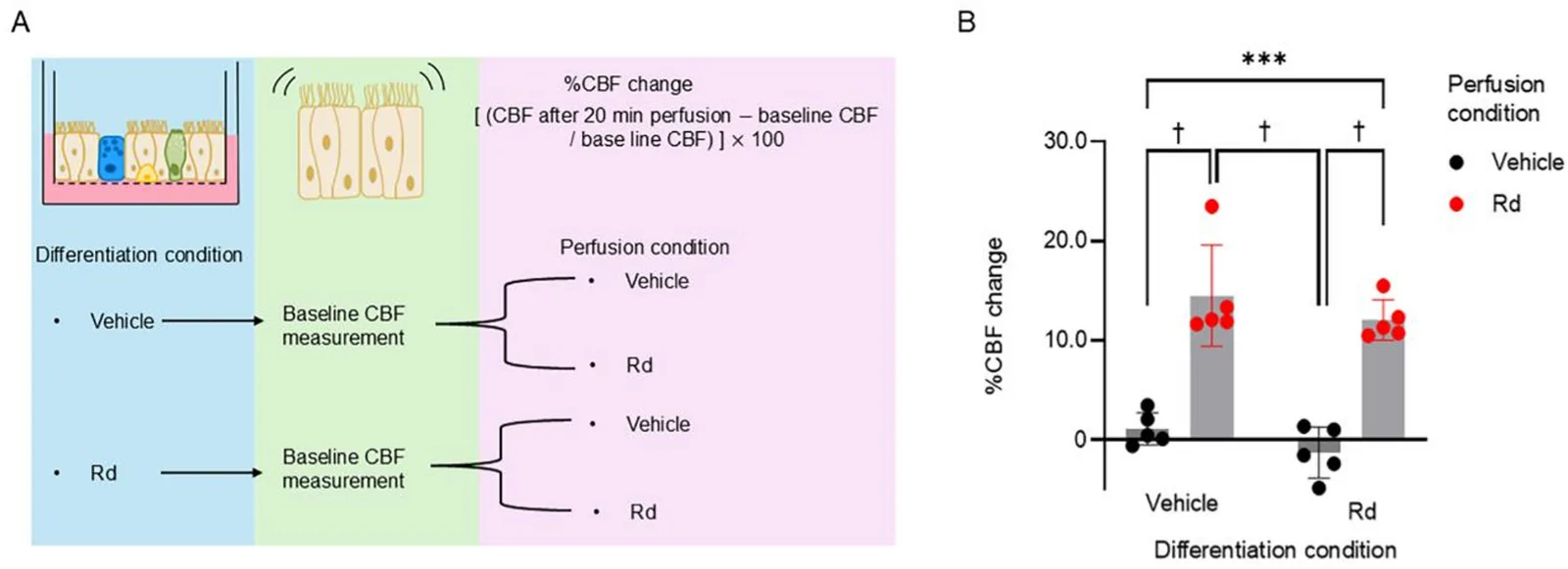
The effects of long-term Rd treatment and additional perfusion treatment on CBF. **A** Schematic representation of the experimental protocol. NHBE cells were differentiated under ALI conditions in the presence or absence of Rd. In both the vehicle-treated and Rd-treated culture groups, CBF was measured using a 2 × 2 experimental design consisting of the vehicle-perfused group and the Rd-perfused group, respectively. **B** CBF was measured under each differentiation and perfusion condition. Data are presented as the mean ± SEM of five independent experiments. Data were analyzed using two-way ANOVA with differentiation condition and perfusion condition as factors. Post hoc comparisons were performed using Tukey’s multiple comparison test. Statistical significance is indicated as ***p < 0.001 and †*p* < 0.0001

We investigated whether long-term Rd treatment during ALI culture affects the baseline CBF of ciliated cells derived from NHBE cells. In both the vehicle-treated and Rd-treated culture groups, CBF was measured using a 2 × 2 experimental design consisting of the vehicle-perfused group and the Rd-perfused group, respectively (Fig. 5A). A two-way ANOVA analysis was performed on the culture and perfusion conditions, and the results showed that the interaction between these factors was not significant. There was no significant difference in baseline CBF between the vehicle- and Rd-treated groups during ALI culture (Fig. 5B). Additional Rd perfusion increased CBF in ciliated cells differentiated in the presence of either vehicle or Rd (Fig. 5B). This suggests that the increase in Ac-α-tubulin-positive cells by long-term Rd treatment and the increase in CBF by short-term Rd treatment may result from different mechanisms of action.

### ERβ inhibition attenuates the effect of Rd on increasing the percentage of Ac-α-tubulin-positive cells

**Fig. 6:**
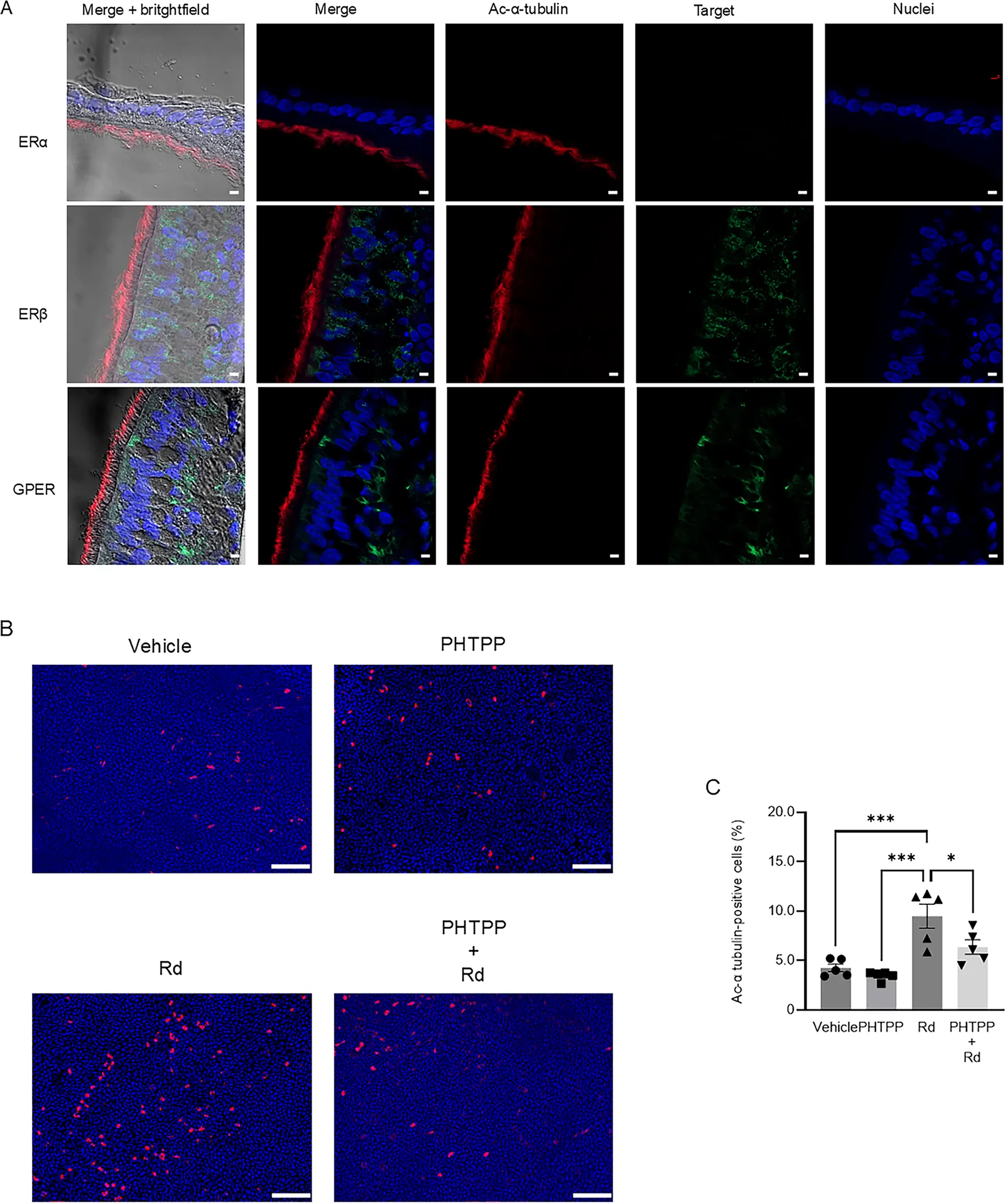

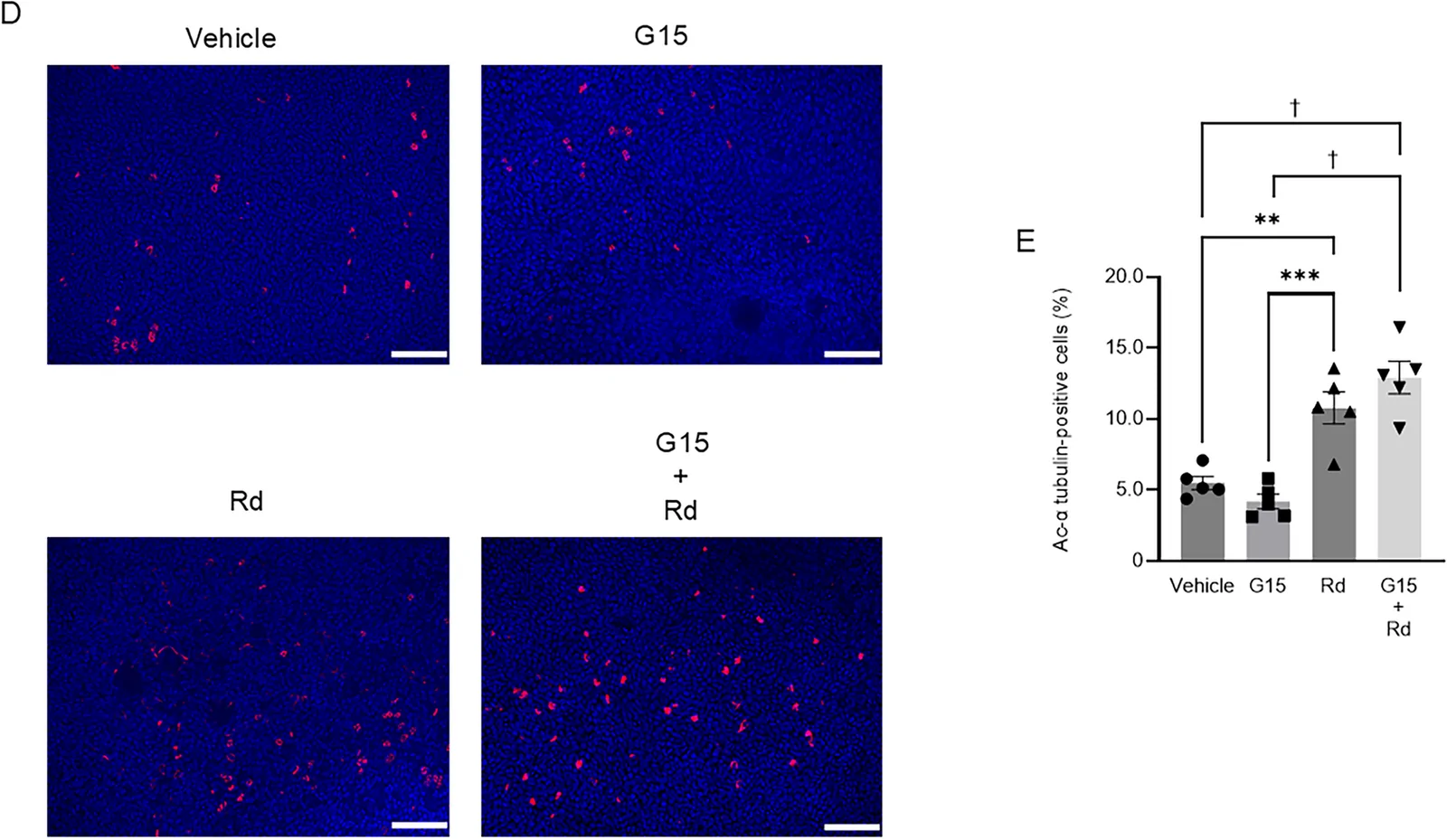
An investigation into the relationship between ER-related signals and Rd-induced increases in Ac-α-tubulin-positive cells. **A** Representative immunofluorescence images of ERα, ERβ, and G protein-coupled estrogen receptor (GPER) in NHBE cells cultured under ALI conditions for 21 days. These images show nuclei stained with Hoechst 33342 (blue), Ac-α-tubulin (red), and the target (green), and merged images that include or exclude bright field. **B**, **D**　Representative immunofluorescence images of NHBE cells treated with vehicle, (B) 10 µM PHTPP, (D) 1 µM G15, and/or 10 µM Rd from the initiation of ALI culture for 10 days. These images show nuclei stained with Hoechst 33342 (blue) and staining for Ac-α-tubulin (red). **C**, **E** Quantification of the percentage of Ac-α-tubulin-positive cells on day 10. Data are presented as the mean ± SEM of five independent experiments. Data were analyzed using one-way ANOVA followed by Tukey’s multiple comparison test. Statistical significance is indicated as *p < 0.05, **p < 0.01, ***p < 0.001, and †*p* < 0.0001. Immunofluorescence images were acquired using a (A) 60× or (B, D) 20× objective lens. Scale bars: (A) 5 μm or (B, D) 100 μm

We hypothesized that ER-related signaling may be involved in the Rd-induced increase in the percentage of Ac-α-tubulin-positive cells in airway epithelial cells. We therefore performed immunofluorescence staining of NHBE cells on day 21 of ALI culture using specific antibodies against ERα, ERβ, and G protein-coupled estrogen receptor 1 (GPER). ERα was not detected, whereas ERβ and GPER were detected (Fig. 6A). We next examined whether pharmacological inhibition of ERβ or GPER affects the Rd response. NHBE cells were treated with the selective ERβ antagonist PHTPP (10 µM) or the selective GPER antagonist G15 (1 µM) in the presence or absence of Rd. PHTPP significantly reduced the percentage of Ac-α-tubulin-positive cells in Rd-treated cells (Fig. 6B, C, and SI 7A). In contrast, G15 did not significantly alter the Rd-induced increase in the percentage of Ac-α-tubulin-positive cells (Fig. 6D, E, and SI 7B). These results provide pharmacological evidence suggesting that ERβ-related signaling, rather than GPER signaling, may contribute to the Rd-induced increase in the percentage of Ac-α-tubulin-positive cells.

### Rd increases FOXJ1 mRNA transcript without significant changes in MCIDAS and HES1 mRNA transcripts

**Fig. 7:**
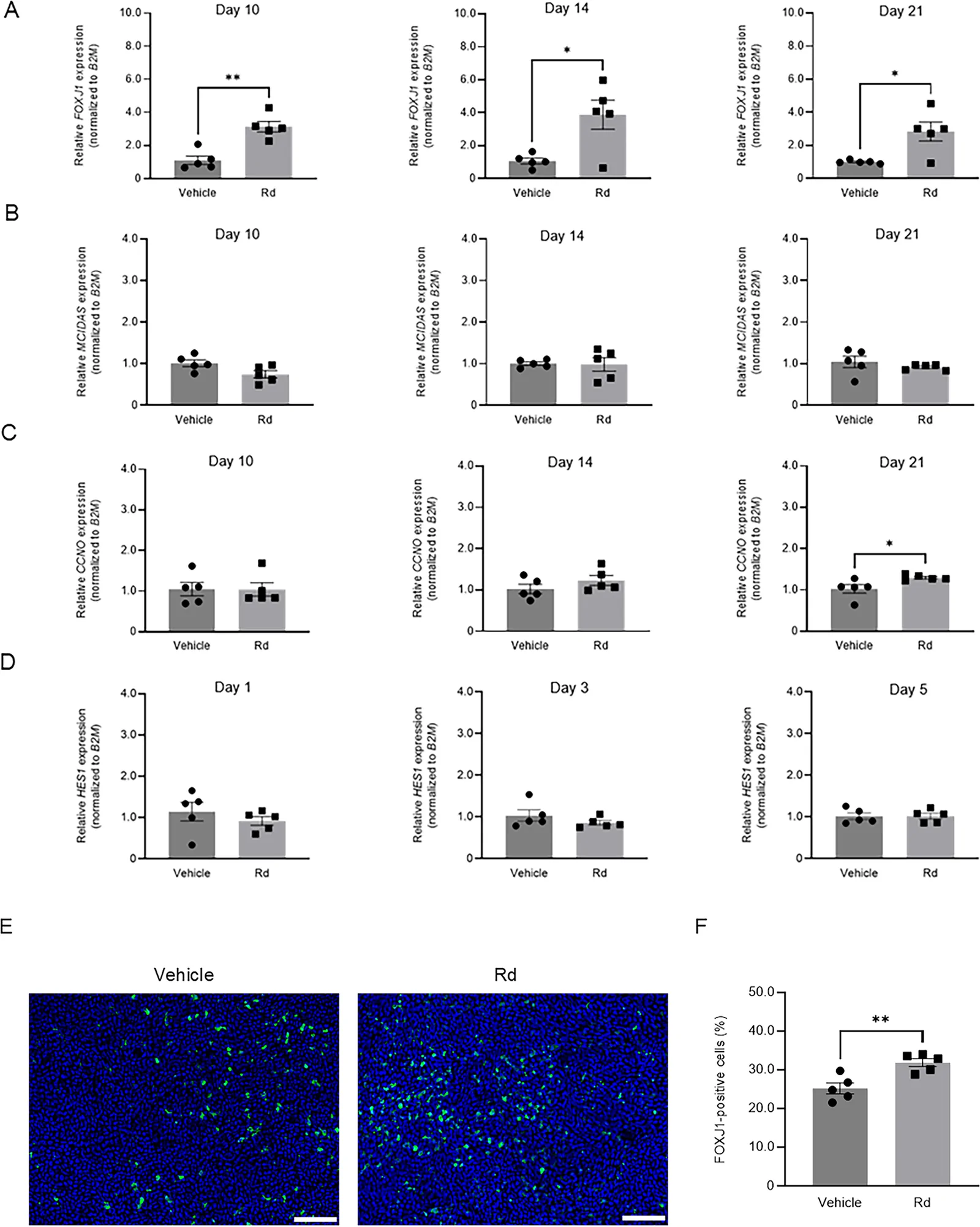
The effect of Rd on ciliated cell differentiation-related genes. **A**–**C** mRNA expression levels of (A) *FOXJ1*, (B) *MCIDAS*, and (C) *CCNO* in NHBE cells treated with vehicle or 10 µM Rd from the initiation of ALI culture for 10, 14, and 21 days. **D** mRNA expression level of *HES1* in NHBE cells treated with vehicle or 10 µM Rd from the initiation of ALI culture for 1, 3, and 5 days. **E** Representative immunofluorescence images of NHBE cells treated with vehicle or 10 µM Rd from the initiation of ALI culture for 14 days. These images show nuclei stained with Hoechst 33342 (blue) and staining for FOXJ1 (green). **F** Quantification of the percentage of FOXJ1-positive cells on day 14. Data are presented as the mean ± SEM of five independent experiments. Data were analyzed using unpaired Student’s t-test. Statistical significance is indicated as *p < 0.05 and **p < 0.01. All immunofluorescence images were acquired using a 20× objective lens. Scale bars: 100 μm

We next examined the effect of Rd on *FOXJ1* mRNA expression after 10, 14, and 21 days of ALI culture. Rd significantly increased *FOXJ1* mRNA expression at all time points examined (Fig. 7A). We then assessed the mRNA expression of *MCIDAS*, an upstream regulator of ciliogenesis, and *CCNO*, which is involved in basal body amplification. Rd did not significantly alter *MCIDAS* expression at any time point (Fig. 7B), whereas *CCNO* expression was significantly increased only on day 21 (Fig. 7C). To further examine the possible involvement of Notch signaling, we analyzed the mRNA expression of *HES1*, a downstream target gene of Notch signaling. Rd did not significantly affect *HES1* expression after 1, 3, and 5 days of ALI culture (Fig. 7D). To confirm the effect of Rd on FOXJ1 at the protein level, we performed immunofluorescence staining on day 14. Rd significantly increased the percentage of FOXJ1-positive cells (Fig. 7E, F, and SI 8A). These results suggest that the Rd-induced increase in Ac-α-tubulin-positive cells is accompanied by increased FOXJ1 expression. However, Rd did not alter *MCIDAS* or *HES1* mRNA expression and increased *CCNO* mRNA expression only at day 21, suggesting that Rd may have relatively little effect on the upstream transcriptional program of ciliated-cell differentiation under these conditions.

### Inhibition of CREB/CBP-dependent transcription attenuates the Rd-induced increase in the percentage of Ac-α-tubulin-positive cells

**Fig. 8:**
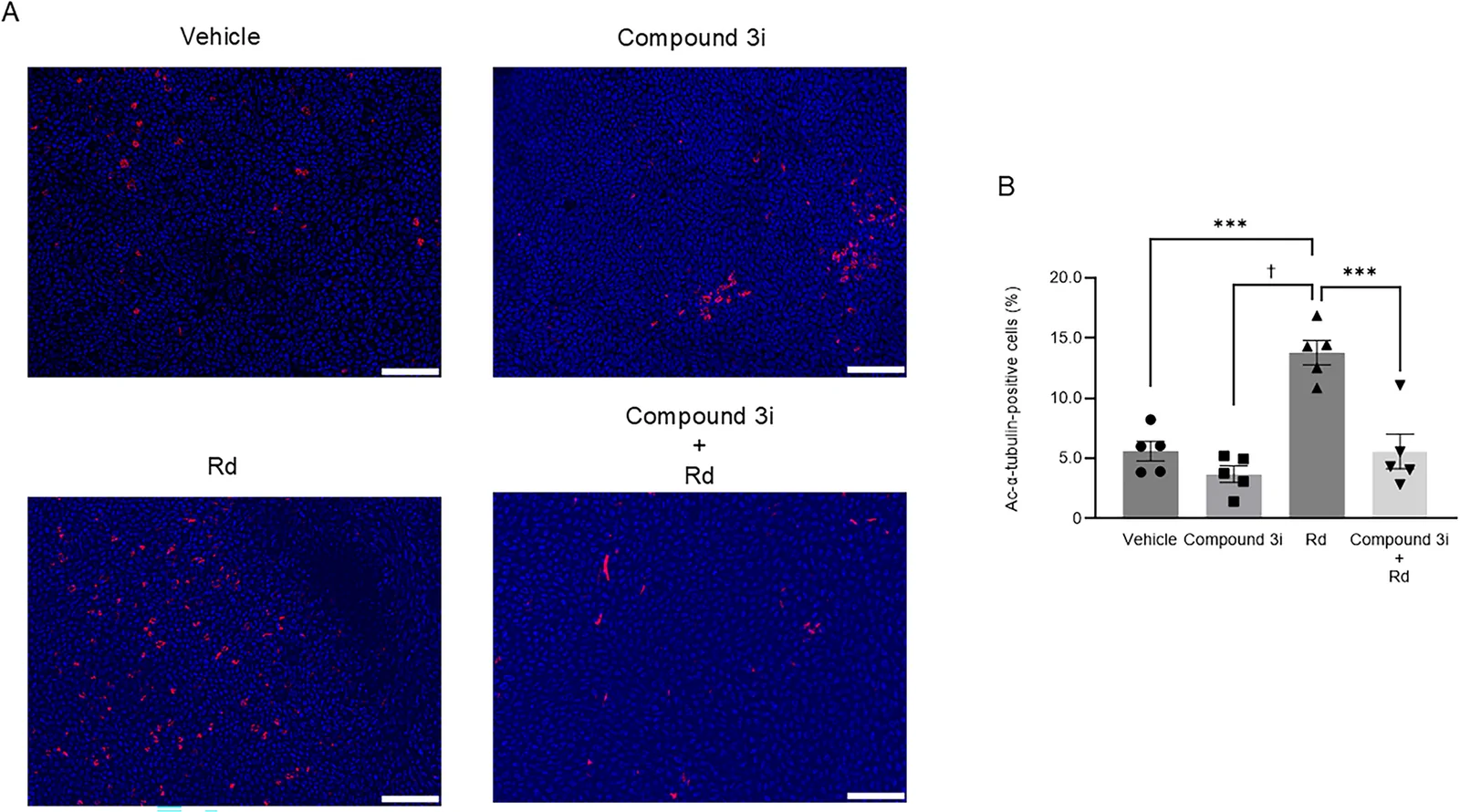
The effect of CREB/CBP-dependent transcription inhibition on the Rd-induced increase in the percentage of Ac-α-tubulin-positive cells. **A** Representative immunofluorescence images of NHBE cells treated with 0.5 µM compound 3i and/or 10 µM Rd from the initiation of ALI culture for 10 days. These images show nuclei stained with Hoechst 33342 (blue) and staining for Ac-α-tubulin (red). **B** Quantification of the percentage of Ac-α-tubulin-positive cells. Data are presented as the mean ± SEM of five independent experiments. Data were analyzed using one-way ANOVA followed by Tukey’s multiple comparison test. Statistical significance is indicated as ***p < 0.001. All immunofluorescence images were acquired using a 20× objective lens. Scale bars: 100 μm

Based on reports that Rd increases cAMP and that CREB is involved in respiratory tract differentiation [12, 15], we hypothesized that Rd may increase the percentage of Ac-α-tubulin-positive cells via CREB, a transcription factor downstream of cAMP signaling. To test this hypothesis, we co-treated NHBE cells with Rd and compound 3i, an inhibitor of CREB/CREB-binding protein (CBP)-dependent transcription, for 10 days beginning at the initiation of ALI culture. Compound 3i (0.5 µM) significantly reduced the Rd-induced increase in the percentage of Ac-α-tubulin-positive cells (Fig. 8A, B, and SI 9A). These results suggest that CREB/CBP-dependent transcription may contribute to the Rd-induced increase in the percentage of Ac-α-tubulin-positive cells.

## Discussion

Airway epithelial homeostasis is maintained by the coordinated function of diverse epithelial cell types. These cell populations are continuously replenished through the differentiation of basal cells, which serve as stem/progenitor cells in the airway epithelium. Disruption of this differentiation program can impair MCC and is one of the characteristic features of chronic airway diseases. In asthma and chronic obstructive pulmonary disease (COPD), MCC is compromised by airway remodeling, including a reduced proportion of ciliated cells and goblet cell metaplasia. Clinically, patients with asthma have been reported to have up to a 1.3-fold higher risk of upper respiratory tract infections and a 2.9-fold higher risk of lower respiratory tract infections than healthy individuals. Patients with COPD also have an approximately 4.8-fold higher risk of pneumonia [24, 25]. Taken together, these findings underscore the importance of both differentiation into ciliated cells and ciliary motility in maintaining respiratory host defense. Although several compounds have been reported to enhance ciliary motility [8], to our knowledge, pharmacological approaches to promote or modulate ciliated epithelial differentiation remain limited. Thus, the present study examined whether Rd modulates the expression of ciliated cell markers in NHBE cells.

Rd increased the percentage of Ac-α-tubulin-positive cells at concentrations up to 10 µM, whereas this effect was attenuated at 30 µM (Fig. 3B–D). Rd at 1–30 µM did not cause any change in the total cell numbers compared to the vehicle under ALI conditions for 10 days (data not shown). The mechanism underlying this non-monotonic response remains unclear. However, it may reflect concentration-dependent differences in receptor engagement, intracellular signaling dynamics, or feedback regulation. Because we previously observed a similar concentration–response pattern for the effect of 17β-estradiol on CBF in murine airway ciliated cells, estrogen receptor-related signaling may be involved in this response. Rd treatment also did not significantly alter LDH release or the number of Hoechst 33342-positive cells (SI 6B, C). Thus, the Rd-induced increase in the percentage of Ac-α-tubulin-positive cells is unlikely to be explained by nonspecific changes in cell viability or cell density. Furthermore, Rd exerts different effects depending on the duration of treatment. Short-term Rd treatment increased CBF (Fig. 5B). Long-term Rd treatment increased the percentage of Ac-α-tubulin-positive cells (Figs. 3 and 4). In this study, the increase in the percentage of Ac-α-tubulin-positive cells by Rd was suppressed by ERβ inhibition (Fig. 6B, C). Sex differences and hormonal changes associated with menopause may affect ciliary motility and mucociliary clearance. Furthermore, CBF is regulated by multiple pathways, including cAMP-dependent, Ca²⁺-dependent, and nitric oxide-related signaling [8], and estrogen receptor-related signaling should also be interpreted within this broader regulatory network.

Rd increased FOXJ1 expression at both the mRNA and protein levels (Fig. 7A, F). In contrast, Rd did not increase *MCIDAS* or *HES1* mRNA expression (Fig. 7B, D), and *CCNO* mRNA expression was significantly increased only on day 21 (Fig. 7C). *CCNO* mRNA expression increased slightly at the later time point. This may reflect an increase in the number of cells that tend to differentiate into ciliated cells due to Rd treatment. Compound 3i attenuated the Rd-induced increase in the percentage of Ac-α-tubulin-positive cells (Fig. 8B). These findings suggest that the Rd mechanism of action may involve CREB/CBP-dependent transcription rather than acting through upstream ciliogenesis-related genes or canonical Notch-HES1 signaling.

This study has several limitations. First, the present study lacks in vivo validation. In the biological environment, airway epithelial differentiation is regulated by multiple niche-derived factors, including EGF, FGF, and cytokines, which may be supplied by stromal cells, endothelial cells, and immune cells [26]. This multicellular airway microenvironment is difficult to fully reproduce in an ALI culture system. Further studies using in vivo models or more complex co-culture systems are therefore warranted. However, there are differences between humans and rodents in terms of the anatomical characteristics and cellular composition of the airways [27, 28]. In mice, basal cells are primarily distributed in the trachea and proximal airways and are poor or almost absent in the distal bronchioles. By contrast, basal cell-containing pseudostratified epithelium extends distally to the terminal bronchioles in humans [29]. Therefore, it is preferable that efficacy be evaluated in the future through clinical trials in humans. On the other hand, a complementary approach that serves as an alternative to clinical trials at the research level is an in vitro model tracking the movement of fluorescent beads on the apical surface of airway epithelial cells [30, 31]. In future studies, it will be necessary to conduct such assays to clarify whether the effects of Rd on Ac-α-tubulin-positive cells and CBF lead to the improvement of mucociliary clearance. Second, although Ac-α-tubulin is a widely used ciliated cell marker, it does not by itself confirm full ciliary maturation, structure, or function. Our findings are supported by increased FOXJ1 expression (Fig. 7A, F) and the responsiveness of differentiated ciliated cells (Figs. 2E and 5B). Additional analyses such as assessment of cilia morphology or mucociliary transport would further clarify the functional significance of Rd-induced changes.

The clinical relevance of Rd also requires consideration of pharmacokinetics and achievable tissue concentrations. In rodents, Rd has been reported to distribute most strongly to the lung after intravascular administration [32, 33]. In addition, a Phase I clinical trial is being conducted to investigate the pharmacokinetics and safety of Rd in humans. In this Phase I trial, single and repeated intravenous administrations are conducted, and Cmax has been reported to range from 3 to 20 µM [34]. Because Rd exerted effects on the percentage of Ac-α-tubulin-positive cells and CBF at 10 µM in the present study and in our previous studies [15], this concentration may potentially have pharmacological relevance. 

In conclusion, Rd increased the percentage of Ac-α-tubulin-positive cells and CBF in ciliated cells derived from NHBE cells. This suggests that Rd may be involved both in increasing CBF through rapid signal pathways and regulating FOXJ1 and Ac-α-tubulin expression. The increase in the percentage of Ac-α-tubulin-positive cells may be associated with FOXJ1 upregulation, CREB/CBP-dependent transcription, and ERβ-related signaling. These findings suggest that Rd possibly modulated the expression of ciliated cell markers, such as acetylated α-tubulin and FOXJ1, and ciliary motility in human airway epithelial cells.

## Supplementary Information

Below is the link to the electronic supplementary material.

Supplementary Material 1

## Data Availability

Data will be made available upon reasonable request.

## Abbreviations

Ac-α-tubulin: Acetylated α-tubulin
ALI: Air-liquid interface
ANOVA: Analysis of variance
BSA: Albumin from bovine serum
CBF: Ciliary beat frequency
CBP: CREB-binding protein
COPD: Chronic obstructive pulmonary disease
CREB: cAMP response element binding protein
DMSO: Dimethyl sulfoxide
ER: Estrogen receptor
FOXJ1: Forkhead box J1
GPER: G protein-coupled estrogen receptor
LDH: Lactate dehydrogenase
MCC: Mucociliary clearance
MCIDAS: Multiciliate differentiation and DNA synthesis-associated cell cycle protein
MUC5AC: Mucin 5, subtype AC
NHBE: Primary normal human bronchial epithelial
PBS: Phosphate-buffered saline
Rd: Ginsenoside Rd
RT-qPCR: Reverse transcription-quantitative PCR
