# Supplementary material for: Ginsenoside Rd promotes the expression of acetylated α-tubulin and FOXJ1 in human bronchial epithelial cells
Source: J Nat Med. 2026 Sep 23;80(6):1722–36. doi: 10.1007/s11418-026-02084-9 (PMC13723605; doi:10.1007/s11418-026-02084-9)
Supplement: Supplementary file 1 [file 11418_2026_2084_MOESM1_ESM.docx]

*Supplementary Information*

***The Journal of Natural medicines***

Ginsenoside Rd promotes differentiation into ciliated cell in human bronchial epithelial cells

Kouta Noriyama^1,*^, Seikou Nakamura^2^, Nobuyo Tamiya^3, 4^, Yuki Toda^1^, Shigekuni Hosogi^1^, Eishi Ashihara^1,†^

^1^Laboratory of Clinical and Translational Physiology, Kyoto Pharmaceutical University, 5 Nakauchi-cho, Misasagi, Yamashina-ku, Kyoto 607–8414, Japan.

^2^Laboratory of Pharmacognosy, Kyoto Pharmaceutical University, 1 shichono-cho, Misasagi, Yamashina-ku, Kyoto 607-8412, Japan.

^3^Pulmonary Medicine, Rakuwakai Otowa Hospital, 2 chinji-cho, Otowa, Yamashina-ku, Kyoto 607-8062, Japan.

^4^Department of Pulmonary Medicine, Graduate School of Medical Science, Kyoto Prefectural University of Medicine, 465 Kajii-cho, Kawaramachi-Hirokoji, Kamigyo-ku, Kyoto 602-8566, Japan.

^†^Present address: Kyoto Red Cross Blood Center, Nakajimakitanokuchi-cho, Fushimi-ku, Kyoto, 612-8451, Japan.

^*^Corresponding authors: kd23007@ms.kyoto-phu.ac.jp; Tel.: +81-75-595-4705

Laboratory of Clinical and Translational Physiology, Kyoto Pharmaceutical University, 5 Nakauchi-cho, Misasagi, Yamashina-ku, Kyoto 607–8414, Japan.

ORCID: 0009-0000-1361-9206

**Materials and methods**

*LDH assay*

Cytotoxicity was assessed by measuring the release of lactate dehydrogenase (LDH) in the basolateral medium using a Cytotoxicity LDH Assay Kit-WST (Wako Pure Chemical Industries, Tokyo, Japan) according to the manufacturer's instructions. Absorbance was measured in a microplate reader (GloMax®, Promega, Madison, WI, USA).

SI 1: Antibodies used in this study.

| Antibody | Host | Source | Identifier |
| --- | --- | --- | --- |
| anti-acetylated tubulin | rabbit | Cell Signaling | 5335S |
| anti-ERα | mouse | Santa Cruz Biotechnology | sc-8005 |
| anti-ERβ | mouse | Santa Cruz Biotechnology | sc-390243 |
| anti-FOXJ1 | mouse | Santa Cruz Biotechnology | sc-53139 |
| anti-GPER | rabbit | Gene Tex | GTX107748 |
| anti-MUC5AC | rabbit | Cell Signaling | 61193 |
| Alexa Fluor 488TM anti-mouse | goat | Thermo Fisher Scientific Inc | A11001 |
| Alexa Fluor 488TM anti-rabbit | goat | Thermo Fisher Scientific Inc | A11008 |
| Alexa Fluor 546TM anti-rabbit | goat | Thermo Fisher Scientific Inc | A11010 |
| Alexa Fluor 647TM anti-rabbit | donkey | Thermo Fisher Scientific Inc | A31571 |

This table shows the antibodies used for immunofluorescent staining in this study. Immunofluorescence staining was performed at a dilution factor of 1:100 for anti-ERα, anti-ERβ, and anti-GPER antibodies or 1:1000 for the other antibodies.

SI 2: Gene-specific primers used in this study.

| Target | Forward sequences (5’ to 3’) | Reverse sequences (5’ to 3’) |
| --- | --- | --- |
| *B2M* | CCAAGATAGTTAAGTGGGATCGAGA | AGCAAGCAAGCAGAATTTGGA |
| *FOXJ1* | TTCTGCTACTTCCGCCACG | TGGTTCGTCCTTCTCCCGA |
| *MCIDAS* | TCGCAGAACCATTCCCACCA | GCGACATCATAGAGGATGAGTCTGA |
| *CCNO* | TCGCTTGCAAACAGGTGGA | AGGGTGAAGTGCAGCTTGTG |
| *HES1* | ACGACACCGGATAAACCAAAGA | ATGCCGCGAGCTATCTTTCT |

This table shows the gene-specific primers used for reverse transcription-quantitative PCR in this study.

**SI 3**

**SI 3.** Immunofluorescence staining of NHBE cells on day 21 under ALI culture conditions

(A, B) Representative immunofluorescence images of NHBE cells cultured for 21 days under ALI conditions. Each image shows a single channel, representing cell nuclei stained with Hoechst 33342 (blue), (A) Ac-α-tubulin (red), and (B) MUC5AC (yellow). All immunofluorescence images were acquired using a 20× objective lens. Scale bars: 100 µm.

**SI 4**

**SI 4.** Immunofluorescence staining of NHBE cells on day 10 under ALI culture conditions with vehicle or Rd treatment

(A, B) Representative immunofluorescence images of NHBE cells treated with vehicle or 1, 3, 10, or 30 µM Rd from the initiation of ALI culture for 10 days. Each image shows a single channel, representing cell nuclei stained with Hoechst 33342 (blue), (A) Ac-α-tubulin (red), and (B) MUC5AC (yellow). All immunofluorescence images were acquired using a 20× objective lens. Scale bars: 100 µm.

SI 5

**SI 5.** Immunofluorescence staining of NHBE cells on day 10 or day 14 under ALI culture conditions with vehicle or Rd treatment

(A, C) Representative immunofluorescence images of NHBE cells treated with vehicle or 10 µM Rd from the initiation of ALI culture for (A) 14 days or (C) 21days. Each image shows a single channel, representing cell nuclei stained with Hoechst 33342 (blue) and Ac-α-tubulin (red). (B, D) Representative immunofluorescence images of NHBE cells treated with vehicle or 10 µM Rd from the initiation of ALI culture for (B) 14 days or (D) 21days. The images show nuclei stained with Hoechst 33342 (blue), MUC5AC (yellow), and their merged image. All immunofluorescence images were acquired using a 20× objective lens. Scale bars: 100 µm.

**SI 6**

**SI 6.** Evaluation of the rate of increase in Ac-α-tubulin-positive cells and cytotoxicity by long-term Rd treatment.

(A) NHBE cells were treated with vehicle or 10 μM Rd from the initiation of ALI culture for 10, 14, and 21 days. Simple linear regression analysis was performed using time-course data from each group. Dashed lines indicate fitted regression lines. Slope analysis showed a significantly greater rate of increase in the percentage of Ac-α-tubulin-positive cells in Rd-treated cells than in Vehicle-treated cells (p = 0.026). (B, C) NHBE cells were treated with vehicle or 10 μM Rd from the initiation of ALI culture for 21 days. (B) LDH release was measured in the basolateral medium. (C) The number of Hoechst33342-positive cells was counted. Data were analyzed using an unpaired Student’s t-test. Data are presented as the mean ± SEM of five independent experiments.

**SI 7**

**SI 7.** Immunofluorescence staining of NHBE cells on day 10 under ALI culture conditions with ER inhibitor and Rd co-treatment

(A, B) Representative immunofluorescence images of NHBE cells treated with vehicle, (A) 10 µM PHTPP, (B) 1 µM G15 and/or 10 µM Rd from the initiation of ALI culture for 10 days. Each image shows a single channel, representing cell nuclei stained with Hoechst 33342 (blue) and Ac-α-tubulin (red). All immunofluorescence images were acquired using a 20× objective lens. Scale bars: 100 µm.

**SI 8**

**SI 8.** Immunofluorescence staining of NHBE cells on day 14 under ALI culture conditions with vehicle or Rd treatment

(A) Representative immunofluorescence images of NHBE cells treated with vehicle or 10 µM Rd from the initiation of ALI culture for 14 days. Each image shows a single channel, representing cell nuclei stained with Hoechst 33342 (blue) and FOXJ1 (green).

**SI 9**

**SI 9.** Immunofluorescence staining of NHBE cells on day 10 under ALI culture conditions with CREB/CBP-dependent transcription inhibitor and Rd co-treatment

(A) Representative immunofluorescence images of NHBE cells treated with vehicle, 0.5 µM compound 3i and/or 10 µM Rd from the initiation of ALI culture for 10 days. Each image shows a single channel, representing cell nuclei stained with Hoechst 33342 (blue) and Ac-α-tubulin (red). All immunofluorescence images were acquired using a 20× objective lens. Scale bars: 100 µm.
